# Immunological Profile of Vasospasm after Subarachnoid Hemorrhage

**DOI:** 10.3390/ijms24108856

**Published:** 2023-05-16

**Authors:** Michele Romoli, Fabrizio Giammello, Maria Giulia Mosconi, Antonio De Mase, Giovanna De Marco, Anna Digiovanni, Antonio Ciacciarelli, Raffaele Ornello, Benedetta Storti

**Affiliations:** 1 Neurology and Stroke Unit, Department of Neuroscience, Bufalini Hospital, 47521 Cesena, Italy; 2 Translational Molecular Medicine and Surgery, Department of Biomedical, Dental Science and Morphological and Functional Images, University of Messina, 98122 Messina, Italy; 3 Emergency and Vascular Medicine, University of Perugia—Santa Maria Della Misericordia Hospital, 06129 Perugia, Italy; 4 Neurology and Stroke Unit, AORN Cardarelli, 80131 Napoli, Italy; 5 Department of Biomedical and NeuroMotor Sciences of Bologna, University of Bologna, 40126 Bologna, Italy; 6 Department of Neuroscience, Imaging and Clinical Sciences, “G. D’Annunzio” University of Chieti-Pescara, 66013 Chieti, Italy; 7 Stroke Unit, Department of Emergency Medicine, University of Roma La Sapienza—Umberto I Hospital, 00161 Rome, Italy; 8 Department of Biotechnological and Applied Clinical Sciences, University of L’Aquila, 67100 L’Aquila, Italy; 9 Cerebrovascular Diseases Unit, Department of Clinical Neurosciences, Fondazione IRCCS Istituto Neurologico Carlo Besta, 20133 Milano, Italy

**Keywords:** subarachnoid hemorrhage, vasospasm, early brain injury, delayed ischemia

## Abstract

Subarachnoid hemorrhage (SAH) carries high mortality and disability rates, which are substantially driven by complications. Early brain injury and vasospasm can happen after SAH and are crucial events to prevent and treat to improve prognosis. In recent decades, immunological mechanisms have been implicated in SAH complications, with both innate and adaptive immunity involved in mechanisms of damage after SAH. The purpose of this review is to summarize the immunological profile of vasospasm, highlighting the potential implementation of biomarkers for its prediction and management. Overall, the kinetics of central nervous system (CNS) immune invasion and soluble factors’ production critically differs between patients developing vasospasm compared to those not experiencing this complication. In particular, in people developing vasospasm, a neutrophil increase develops in the first minutes to days and pairs with a mild depletion of CD45+ lymphocytes. Cytokine production is boosted early on after SAH, and a steep increase in interleukin-6, metalloproteinase-9 and vascular endothelial growth factor (VEGF) anticipates the development of vasospasm after SAH. We also highlight the role of microglia and the potential influence of genetic polymorphism in the development of vasospasm and SAH-related complications.

## 1. Background

Subarachnoid hemorrhage (SAH) represents a catastrophic event, associated with high mortality and poor rates of full recovery [1]. Most SAH cases are related to the rupture of an aneurysm in the main branches of intracerebral arteries, a condition which might be suitable for emergent intervention via clipping or coiling procedures [2]. Case fatality had a yearly 0.9% decline over the past two decades, mainly in relation to timely diagnosis and significant improvements in devices [1,3]. Despite optimal management, however, the rates of good functional recovery remain unsatisfactory. Studies from high-income countries report rates of death as high as 25%, with almost two thirds of patients left with neurological deficit with some impact on daily activities. Such outcomes weigh heavy on SAH patients, which are on average 20 years younger than people suffering from ischemic stroke, therefore translating into a longer burden of chronicity, disability and cost to society [1].

After the development of coiling, which was associated with a reduction in the fatal SAH rate [4], a further attempt to mitigate the risk of poor neurological outcome after SAH came from the management of vasospasm [5,6,7]. Vasospasm can develop in two-thirds of people with aneurysmal SAH, with several factors implied in its pathophysiology [8,9,10,11]. Hemoglobin, its degradation products and erythrocytes can elicit local endothelial reactions, leading to the narrowing of arteries next to the site of the aneurysm, and to potential ischemia. Counting on the association between vasospasm, delayed cerebral ischemia (DCI) and poor functional outcome, landmark studies were performed, which proposed nimodipine as a potential tool to limit angiographic vasospasm and improve the overall prognosis [5]. Over the following decades, nimodipine was confirmed as the only drug able to improve the neurological outcome, but it failed to be consistently associated with a control of vasospasm [6], putting the consequentialist logic of vasospasm-to-DCI under debate. Agents aimed at controlling endothelial factors also failed to mitigate vasospasm or mortality [7], and vasospasm control failed to translate into consistent reduction in mortality [6], further supporting research into complementary mechanisms. Indeed, two-thirds of SAH patients develop vasospasm, but only one-third then develops DCI, implying that other mechanisms should take part in the process [8,9,10,11].

As DCI and vasospasm can happen in a relatively wide temporal window, 3 to 14 days after SAH, research has focused on early events that may pave the way to complications with negative impacts on the outcome. The acute effects of subarachnoid blood and the transient ischemia that may accompany aneurysm rupture (referred to as early brain injury, EBI) can already develop in the first 72 h [12]. Despite its definition being elusive, and with neuroimaging, clinical and biomarker criteria applied with poor consistency even across studies, EBI seems to represent a key determinant for poor prognosis in the long term [12]. Therefore, what happens in the very early phase seems to produce events and have an impact way beyond the first days after SAH.

The inflammatory response after SAH rolls out in two main phases. At SAH onset, blood extravasation leads to the invasion of the CSN by unselected white cells. After this initial step, the activation of the immune system can induce the secretion of cytokines and other factors able to promote a targeted immune reaction. Microglia and dendritic cell activation, immune cell chemotaxis and cytokine storm develop, with direct consequences in terms of microvascular architecture, vasospasm, early brain injury and eventually long-term prognosis [10,13,14]. Here, we review the immunological profile of SAH, with particular regard to vasospasm, EBI and potential therapeutic targets.

## 2. Inflammatory Response after SAH

DCI emerged as the main cause of mortality and morbidity following SAH [2,15]. DCI was progressively disentangled from vasospasm, with the latter increasing the risk of the former, but with DCI also happening outside of vasospastic mechanisms and vasospasm also developing without leading to DCI [1]. As EBI has been progressively tied to DCI [16], the main mechanisms driving EBI were also investigated in regard to potential windows to treatment.

EBI mechanisms include altered cerebral perfusion induced by increased intracranial pressure (ICP), the imbalance of membrane polarization with cortical spreading depression and uncoupling between pro-inflammatory and immunomodulatory mechanisms [15,16]. The immune system seems to be directly involved in all of these steps, therefore supporting its transversal potential as a therapeutic target.

The increase in ICP leads to a reduction in cerebral perfusion and to a disruption of the blood–brain barrier (BBB), in turn driving leakage, edema and peripheral immune cell access to the subarachnoid space [14]. SAH has a direct impact on the cellularity of the choroid plexus, with a significant increase in the number of several macrophage types shown in animal models [17]. However, SAH does not only concern blood extravasation. Indeed, an increase in ICP itself can induce an immune reaction and proliferation in the epiplexus cells of the choroid plexus [17], a finding that is in line with the hypothesis that CSF secretion can also participate in hydrocephalus in SAH [18].

Cortical spreading depression (CSD), due to abnormal membrane polarization, can happen after SAH and is associated with EBI [1,19]. Among other mechanisms, CSD also has an impact on the immune system. Through caspase-1 activation and the neuronal release of high-mobility group box 1 (HMGB1) and nuclear factor kappa B (NF-κB), CSD can induce a parenchymal inflammatory response, while also inducing a meningeal response with the activation of macrophages and cytokine secretion [20,21,22]. Reactive astrocytosis seems to be a key player in this context, with CSD being tied to an increase in the expression of pro-inflammatory markers, cytokines (IL-6 and IL-1β) and Toll-like receptors (TLR3 and TLR4) [22]. Changes in astrocytic Ca^2+^ levels after SAH have also been demonstrated to alter neurovascular coupling, driving the response toward vasoconstriction [23]. This might link CSD and vasospasm, as clusters of spreading depolarization have also been associated with the formation of sulcal clots [24].

The inflammatory response after SAH rolls out in two stages, with the initial invasion of CNS by blood and immune cells then leading to progressive changes in CNS and systemic inflammatory markers. An increase in pro-inflammatory markers in plasma has been widely demonstrated after SAH, particularly for tumor necrosis factor (TNF), interleukin-1 (IL-1), matrix metallopeptidase 9 (MMP-9) and interleukin-6 (IL-6) [16,25,26]. These changes are also coupled with a decrease in circulating anti-inflammatory cytokines, such as interleukin-10 (IL-10), and an alteration in immune cell function, including impaired phagocytic activity [16,27]. All these phenomena configure a systemic inflammatory response to SAH, the intensity of which seems to correlate with clinical severity: cytokine levels peak with poor-grade hemorrhage, lower cerebral perfusion and broader cerebral edema [12].

Such an immune reaction, however, fails to shield from infective complications of SAH and hospitalization, representing an SAH paradox. Indeed, what has been shown by recent research is that SAH also induces a transient immunosuppression state, with direct implications for the risk of infections during hospitalization. The incidence of pneumonia, urinary tract infections and meningitis is significantly increased in SAH patients [25,28]. SAH induces lymphopenia, with a net decrease in CD4+ and CD8+ T cells, as well as natural killer (NK) cells, in peripheral blood [28,29]. However, CD69+ T-helper cells have been shown to increase in CSF, supporting an activation status soon after SAH, with a peculiar CNS-specific distribution [29]. CD4+ T-helpers also increase during EBI, supporting their value in monitoring the risk of complications [30]. In plasma, Tregs were also shown to be increased during both the EBI and DCI stages, with permanently activated Tregs growing in cases of vasospasm from the EBI stage to the after-EBI stage. Such an increase induced a Th17/Treg imbalance at the systemic level, with a lower ratio during the EBI and DBI phases compared to healthy controls, suggesting a modulation of the systemic environment towards tolerability [30]. On the contrary, in CSF, Th17 cells were shown to be more prevalent compared to peripheral blood during the early and late stages after SAH. The percentage of Treg cells was lower in CSF than in plasma, therefore supporting an opposed Th17/Treg imbalance and fostering inflammation, particularly in early SAH stages [31]. This pairs with the observation of increased IL-17 levels in early SAH stages [31,32] and sheds the light on the search for a main producer of IL-17 during early SAH stages, which might therefore represent a target for intervention such as the expansion of Tregs in early phases [33]. In this regard, the fact that resident T cells in the aneurysm arterial wall can develop to become CD4+ IL-17 cells soon after SAH seems to support the need to look further into differences between aneurysmal vs. non-aneurysmal SAH.

## 3. Kinetics of CSF Cellularity

**Figure 1:**
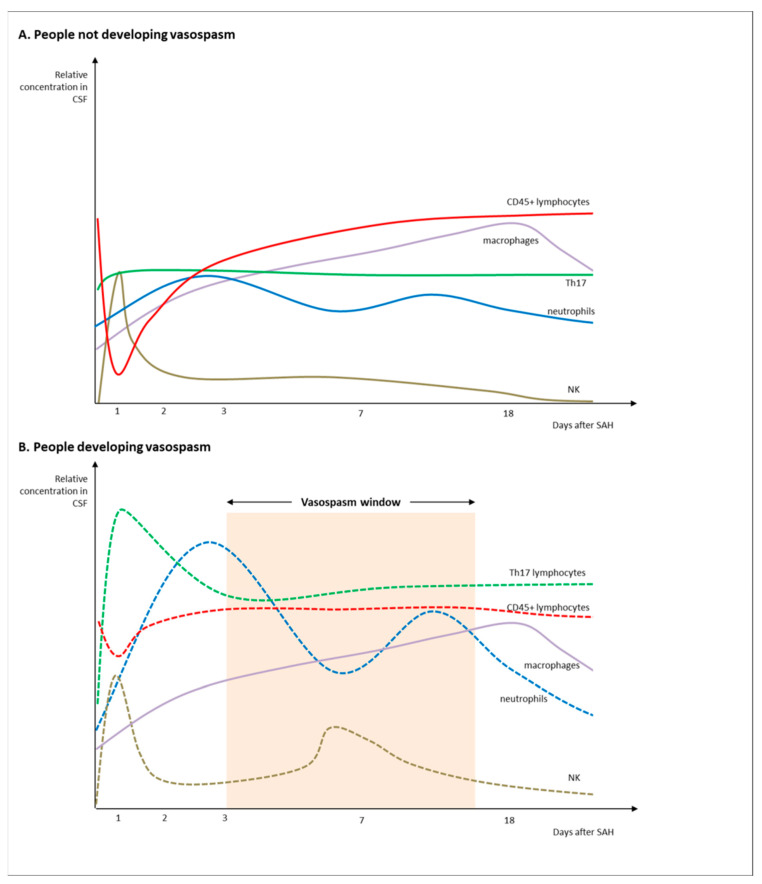
Patterns of CSF cellularity in SAH cases without vasospasm (**A**) and in people developing vasospasm after SAH (**B**).

The interaction between adaptive and innate immunity becomes crucial after SAH. As what happens in very early phases is tied to EBI, which in turn correlates with long-term prognosis, CSF cellularity has gained significant importance, particularly when investigated for evolution in time and space (Figure 1).

The activation of innate immunity has been detected immediately after blood extravasation, with a significant accumulation of neutrophils happening as early as 10 min after SAH [34]. Such an invasion is not limited to microvasculature but also involves brain parenchyma, with cross-talk between neutrophils and microglia thought to play a significant role in the development of complications [35]. In animal models, the concentration of neutrophils immediately after SAH correlated with poor prognosis and vasospasm [13,36,37]. Such a direct relationship was also reported in a cohort study enrolling 236 patients with aneurysmal SAH, with a relative increase in neutrophils in CSF over the first three days, together with a relative decrease in lymphocytes. Neutrophil percentage emerged as an independent predictor of vasospasm even after adjustment for Fisher CT grade, Hunt and Hess score, treatment strategy and age [38]. Results also suggested higher levels of neutrophil-expressed peroxidase in CSF, supporting the concept that neutrophils themselves build a pro-inflammatory environment already in the first three days, potentially paving the way for the development of vasospasm later on [38]. Further reinforcing the role of neutrophil-driven inflammation, the neutrophil/lymphocyte ratio emerged as a potential tool to predict worse prognosis and also stratify patients for their risk of DCI and rebleeding [39,40]. In line with this hypothesis, the early blockage or limitation of neutrophil activity after SAH in animal models translates into preserved arteriolar function, the prevention of vasospasm and higher neurobehavioral recovery [34,36].

Beyond neutrophils, other immune cells are involved after SAH, with the reaction rolling out over time and space. The increase in leukocyte after SAH, which peaks between day 3 and 6, also includes monocytes and macrophages [38,41,42]. Together with lymphocyte count, monocytes and macrophages tend to increase over time, peaking at around day 17, with a rather progressive build up over time that also includes a transition to a higher number of siderophages in later stages [43]. Clinical studies support the concept that macrophages have a crucial role in all SAH stages, from rupture to complications. In SAH patients, macrophage infiltration was associated with smooth muscle cell proliferation and disorganized wall thickening already in the hyperacute stage, less than 12 h from rupture [44]. Monocyte infiltration, assessed with multicolor flow cytometry, was also shown to predict the occurrence of DCI. In particular, infiltrating monocytes transitioned from a non-classical phenotype (CD14dim CD16+) to a CD163+ or CD69+ status, implying CSN activation and evolution towards hemosiderophages. Such a transition is stimulated by inflammatory chemokines (CXCL1, CXCL9, CXCL10 and CXCL11) and specific molecules such as monocyte chemoattractant protein-1, and it seems to happen rather exclusively in CNS, while peripheral blood mainly sees changes in T-cell composition [45,46]. CD163, the high-affinity scavenger receptor for the hemoglobin–haptoglobin complex, progressively gains expression in macrophages, also highlighting a transition to the M2 subtype over time. Such a shift, however, is again exclusive of CNS macrophages, not appearing in plasma [45]. Preclinical studies further confirm the role of macrophages in driving vasospasm, with the depletion of myeloid cells before SAH also being found to improve the rates and features of vasospasm after SAH [35].

Innate immunity can also take part in SAH with NK cells. In a small sample study, the monitoring of NK cells in CNS revealed significant variations over time after SAH. In particular, NK cells accumulated in the CSF after SAH progressively, from day 1 to day 6, with a subsequent decrease. The number of activated NKs expressing CD6 and CD56 for a cytotoxic phenotype directly correlated with the risk of developing vasospasm, but also with the grade of vasospasm, DCI and SAH grading [47].

Focusing on adaptive immunity, evidence on the presence and possible role of T-cell activation in the CNS after SAH date back to the 1990s. In a small sample of 10 patients, a higher number of CD8+ cells was reported in the CSF of patients developing DCI between day 3 and day 8, suggesting a somewhat delayed increase compared to innate immunity cells [48]. Nevertheless, the role played by lymphocytes, the prominent cell type of adaptive immunity, is still far from being completely cleared, and actual evidence is limited and controversial. A recent study investigating flow cytometry on CSF samples longitudinally collected after SAH showed a remarkable reduction in the CD45+ cells in SAH patients when compared to controls in the hyperacute phase (days 0–1) [26]. Such “hyperacute depletion” of CD45+ cells was more profound in people not developing vasospasm, leading to the hypothesis that a lack of immunomodulation fostered a proinflammatory state, leading to vasospasm. Following the steep decrease, a gradual increase in CD45+ count in CSF was seen at all sequent timepoints, peaking on day 10 after SAH. In the acute phase, there was also an higher number, when compared to controls, of CD3-CD161+ NK cells, and an even more remarkable increase in CD8 + CD161 + Th17 cells. Both cells counts abruptly decreased in the following days. Patients who developed VSP showed, in the acute phase, an even higher number of CD8 + CD161 + Th17 cells, known to secrete IL-17, which increases the local production of chemokines, recruits monocytes and has been shown to contribute to inflammation in several disease models [31]. Th17 cells have been found in the arterial wall of unruptured aneurysms, therefore potentially taking part from the very first SAH stages to the remodeling of CNS environment, which may represent the common pathway toward vasospasm and DCI [26] (Table 1).

The driver for adaptive immunity response is still uncertain, as several drivers can at least take part. In addition to cytokine storm and immune cell-to-immune cell activation, however, a marginal action could also be played by arachnoid cells. Indeed, these layer cells, previously thought to be inert from an immune perspective, can serve as antigen-presenting cells (APCs) when stimulated by blood. Therefore, in SAH, they may be able to directly activate T lymphocytes, particularly in a subacute stage (5–6 days), when an increase in IL-2r, a hallmark of lymphocyte activation, can be demonstrated [49].

## 4. Microglia Involvement in SAH and Vasospasm

Microglia include the resident immune cells of the central nervous system and can play a double-edged role in neuro-inflammation, which is associated with the functional outcome of SAH. The fact that microglia are able to self-renew makes them completely different from sibling myeloid-lineage cells such as meningeal macrophages and CSF monocytes [50].

Noxious stimuli can activate microglia on a spectrum ranging from two complementary phenotypes, namely M1 and M2. M1 is a pro-inflammatory phenotype associated with the secretion of interleukin-1β (IL-1β), interleukin-6 (IL-6), tumor necrosis factor-α (TNF-α) and inducible nitric oxide synthase (iNOS), while M2 is anti-inflammatory and produces transforming growth factor β (TGF-β), interleukin-10 (IL-10) and CD206 [51].

As for other immune cells, the response of microglia after SAH evolves across time and space. After SAH, the extravasation of blood following aneurysm rupture induces a strong inflammatory response associated with the activation of microglia [10]. The number of microglial cells steeply increases already at 5–8 days and peaks at 9–15 days after the event [52]. Microglia with M1 and M2-prevalent phenotypes often coexist. M1 microglial cells predominate during the early stage of inflammation, playing a role in brain injury, while M2 microglia mostly appear in the late stage of inflammation, exerting a protective role [53]. More in detail, the expression of M1 markers such as interleukin-6 (IL-6), tumor necrosis factor (TNF) and Toll-like receptor 4 (TLR4) predominates during the first few days [54], while 5–10 days after the event, the up-regulation of IL-4 and transforming growth factor-beta (TGF-β) favors the transition to the M2-predominant phenotype [26,50].

The wave of response also evolves in space. Indeed, a microglial reaction happens in the parenchyma, while blood extravasation is limited to the subarachnoid space. Therefore, microglia respond through the parenchyma to factors outside of the parenchyma. The increase in microglia activation develops from the base to the cortex of both hemispheres, and a rather site-specific reaction consisting of stronger activation close to the clot, aneurysmal rupture and leak site [52]. The microglia reaction seems higher at outer cortical site, with a diminishing gradient going deeper in the cortex. Therefore, if blood is the main driver of such a reaction, strategies to remove or divert it from the subarachnoid space would help in dampening the microglia reaction [55].

There is a relationship between microglia and vasospasm in SAH that is mediated by TLR4, on which heme acts as an agonist [56,57]. TLR4 and microglia contribute to the pathogenesis of cerebral vasospasm through the production of tumor necrosis alpha (TNF), which in turn induces the release of vasoconstrictors [58,59]. As proof of the role of microglia in inducing vasospasm, the depletion of microglia in experimental SAH led to a beneficial effect on cerebral vasospasm and hippocampal neuronal survival [60]. The M1 response is also accompanied by the release of reactive oxygen species that directly damage the cerebral parenchyma and by the further release of inflammatory cytokines which increase the permeability of the blood–brain barrier and promote edema [53,61].

The transition of microglia across two potential states—either detrimental or protective—makes it an interesting therapeutic target for SAH-related neuroinflammation and vasospasm. Indeed, microglia play an important role in hematoma resolution and brain tissue repair. If exposed to carbon monoxide, microglia are able to express heme-oxygenase and to present the CD36 scavenger receptor on the cell surface, which facilitate the phagocytosis of red blood cells and apoptotic neurons and the rapid resolution of hemorrhagic lesions [62]. Inhibiting this transformation of microglia by blocking the function of heme-oxygenase or the expression of CD36 induces a state of reduced microglia activation with consequent worsened outcomes of experimental SAH. Mice in which heme-oxygenase function was inhibited showed a significantly greater vasospasm response and neuronal apoptosis with spatial memory function impairment compared with that seen in control chimeric mice [63]. Some agents useful in contrasting SAH-related vasospasm, including erythropoietin, can also stimulate microglia polarization toward the M2-predominant phenotype and inhibit the release of pro-inflammatory cytokines from microglia [64,65].

## 5. Drivers and Mediators of Immune Reaction at CNS Site

The cellular components of innate immunity have been found in high numbers and in highly activated states in the CSF after SAH, including neutrophils, classical monocytes, and activated microglia and macrophages. Who drives and sustain immune response? What is innate immunity responding to after SAH?

### 5.1. Cytokine Kinetic

**Figure 2:**
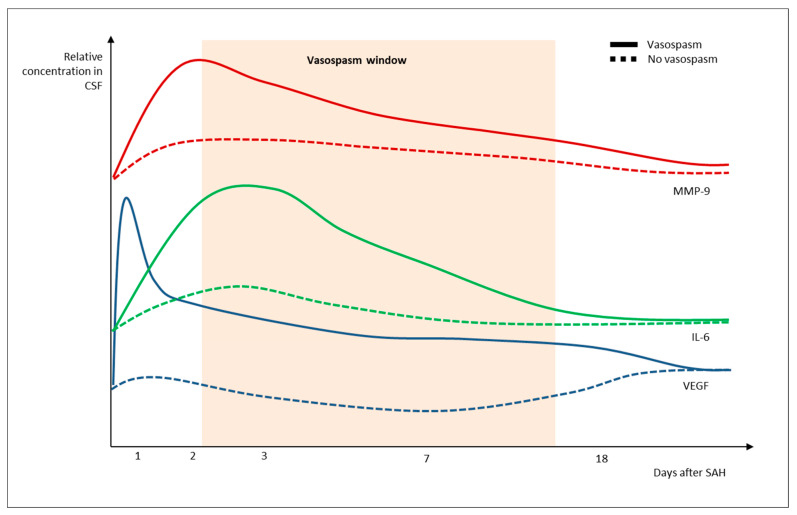
Patterns of IL-6, VEGF and MMP-9 variations after SAH in people with vs. without vasospasm.

The levels of IL-6, IL-7, IL-16, serum amyloid A (SAA), vascular cell adhesion molecule (VCAM)-1, interferon-gamma (IFN-γ), vascular endothelial growth factor (VEGF), basic fibroblast growth factor (bFGF), interferon-inducible protein (IP)-10, monocyte chemoattractant protein-1 (MCP-1), MCP-4 and vascular endothelial growth factor receptor 1/ Fms Related Receptor Tyrosine Kinase 1 (VEGFR-1/Flt-1) all vary significantly in time [26,45,66]. Cytokine expression reveals a distinct pattern of activation over time in SAH (Figure 2).

IL-6 has been proposed as a key mediator of SAH complications. In an observational study enrolling 35 patients with SAH, IL-6 and IL-1b were both found to be increased already 24 h after SAH in people that would then develop vasospasm vs. those without. Such an increase reverberated across the first 9 days, with a slight decline in the following period, suggesting that the pro-inflammatory effect of IL-6 is sustained across time [67]. In a small longitudinal study enrolling 13 patients with SAH, CSF IL-6 levels at SAH onset and at 2–4 days anticipated the occurrence of vasospasm [26]. Beyond vasospasm, plasmatic IL-6 was also found to predict deterioration and seizures in people with SAH and to predict and anticipate the development of DCI when increasing later on after SAH [68]. Stable high levels of IL-6 were also correlated with the development of DCI in a large observational study on 179 SAH patients, with a “no-decrease” IL-6 pattern being associated with doubling the risk of DCI [69]. Similar results were found in humans and animal models, with IL-6 expression shown to peak at day 3, to be increased in aneurysmal blood and CSF as well, and to substantially drive the development of vasospasm [70]. These studies support the investigation of IL-6 as a potential tool to anticipate and eventually mitigate the development of vasospasm and brain injury. To this extent, the modulation of the IL-6 pathway in animal models either with knockout or IL-6 blockade resulted in critically lower rates of vasospasm and neuronal death [70]. IL-1 receptor antagonists have been shown able to reduce IL-6 expression in a phase II clinical study and may result in higher rates of clinical recovery after SAH [71].

Beyond IL-6, other soluble factors have been shown to participate in the immune reaction underlying SAH complications. IL-8 has been shown to mimic the variations in IL-6, with a substantial increase in CSF compared to plasma after SAH, highlighting once more the peculiar spatial activation at the CSN site [72]. In animal models, increased levels of TNF-α are also associated with wider parenchymal damage and poorer outcomes [73]. In a monocentric retrospective study, after propensity-score matching for confounding variables, IL-2, IL-6, IL-8, IL-10 and TNF-α levels correlated with DCI, poor prognosis and mRS recovery [74]. These results, paired with the finding of a persistent cerebral edema in SAH patients with higher levels of TNF-α, support the concept of an ongoing immune reaction at the bleeding site which fosters vasospasm and DCI and limits functional recovery [75].

### 5.2. Metalloproteinase-9 and Vascular Endothelial Factor

The cell membrane and extracellular matrix of the BBB expose several molecules targeted for degradation by the type IV collagenase MMP-9. MMP-9 is reported to have a critical role in the pathophysiology of BBB damage and secondary cerebral edema in ischemic SAH [76]. An elevation in serum MMP-9 levels and/or activity has been described both in animal and in human models of SAH [77,78,79,80]. They have also been reported as an independent predictor of vasospasm, EBI and DCI several days before ischemic deficits, making it a potential biomarker to guide diagnostics and aggressive prophylactic intervention [77,81]. As MMP-9 seems to drive complications after SAH, experimental knockout in a preclinical model has been shown to consistently limit BBB disruption, brain swelling and neurological deficits after SAH, supporting it as a potential target for treatment [76]. Therefore, the question moves to where MMP-9 comes from.

The main mechanism of MMP-9 production after SAH remains unclear, but several mechanisms seem involved in its up-regulation. The endogenous transcription of MMP-9 is regulated via its promoter binding site to NF-κB [82]. Exendin-4 (Ex-4), a glucagon-like peptide 1-receptor (GLP-1R) agonist, approved for adult type 2 diabetes treatment, has been shown to be protective towards BBB integrity by inhibiting MMP-9 in a rat model of ischemic stroke [83]. Moreover, a recent study’s results reported that Ex-4 preserve the BBB integrity through GLP-1R/AMPK-dependent NF-κB/MMP-9 inhibition after SAH, a pathway that seems worthy of further investigation [84].

Another described mechanism is MMP-9 production by reactive astrocytes via the NDRG2-PPM1A pathway. N-myc downstream-regulated gene 2 (NDRG2) is an astrocyte-specific gene which controls apoptosis, astrogliosis and BBB integrity in astrocytes. Specifically, it is a stress-response gene whose expression is up-regulated in cerebral ischemia, trauma and meningiomas, and it up-regulates the expression of MMP-9, with enhanced BBB damage [85]. A recent study reports that MMP-9 is primarily derived from reactive astrocytes peri-BBB in the early stage after SAH and NDRG2 knockout and causes the inhibition of reactive astrocytes and reduced MMP-9 expression. Specifically, NDRG2 directly binds with a Smad-specific protein phosphatase, Mg2+/Mn2+-dependent 1A (PPM1A) and so increases MMP-9 astrocytic expression. The NDRG2-PPM1A interaction has been reported as a potential therapeutic target: QFNP12 is a peptide which mimics PPM1A and competitively binds to NDRG2 with a protective role in the BBB [86].

Several apoptotic pathways are considered to play a role in SAH pathophysiology: the death-receptor pathway, caspase-dependent and independent pathways, and the mitochondrial pathway [87]. An MMP-9 up-regulation secondary to repeated insults has been detected in neurons and endothelial cells which finally died, via “anoikis” (cells’ detachment from their matrix and death) due to laminin degradation [87,88]. Minocycline, a semi-synthetic tetracycline with anti-inflammatory and anti-apoptotic properties, inhibits MMP-9 activity and has shown neuroprotection in cerebral ischemia and in other models of brain injury [87,89,90]. Recent RCT results report that minocycline reduces BBB disruption after an SAH, as evidenced by lower permeability indices; however, a significant reduction in MMP-9 levels is absent [91].

MMP-9 production is also up-regulated by thrombin [92]. Nafamostat, a serin protease inhibitor with endothelial protective effects, similar to argatroban as a direct thrombin inhibitor, has been shown to have protective effects in regard to vasospasm after SAH [93]. In animal models, when administered in vivo, nafamostat promoted endothelial protection from thrombin and hypoxia and caused a reduction in MMP-9 levels, corresponding to improved functional outcome after SAH [94].

MMP-9 expression is also regulated by the mitogen-activated protein kinase (MAPK) pathway, known to also impact cell proliferation, differentiation and apoptosis. The MAPK pathway, including the extracellular-signal-regulated kinase (ERK) and p38 protein, has been implicated in the pathophysiology of SAH, DCI and vasospasm. SAH can induce phosphorylation changes in p38 MAPK and ERK as a first reaction after SAH, already hours after SAH [95]. Such changes in turn result in a consistent increase in the transcription of genes involved in SAH-related inflammatory response, including endothelin and IL-6 [95,96]. Moreover, the MAPK pathway has been directly associated with the development of vasospasm, with MAPK pathway inhibition resulting in substantial reductions in inflammatory cytokines, lower MMP-9 levels, a preserved function of cerebral arteries and improved neurological function [96,97].

Besides MMP-9, the concentration of vascular mitogens (vascular endothelial growth factor, VEGF; platelet-derived vascular growth factors, PDGFs) is also increased during vasospasm after SAH, probably as direct consequence of endothelial damage [98]. In animal models and humans, the development of vasospasm has been anticipated by a consistent increase in serum von Willebrand factor (vWF), MMP-9, and VEGF levels [26,99]. In a clinical study, the evolution of CSF levels of VEGF and MMP-9 was shown to critically diverge between people developing vasospasm vs. those who did not, with the former facing higher levels of both molecules in the very early phase, and levels being still high in the days preceding vasospasm [26]. High levels of VEGF and VEGFR-1/Flt-1 in early stages correlated with the occurrence of vasospasm and poor recovery after SAH in clinical monitoring studies [13].

As MMP-9 and VEGF can interact and increase neuroinflammation, the increase in IL-6 seems to represent a further step in the creation of a critically pro-inflammatory state at the bleeding site [26,45,80,100]. As a key determinant of vascular proliferation and wall thickening, VEGF has been targeted as a potential contributor to EBI and vasospasm. Bevacizumab, an anti-VEGF antibody, has been reported to reduce cerebral vasospasm and DCI thanks to its capacity to attenuate VEGF-stimulated angiogenesis and vascular cell proliferation [101]. As anti-VEGF treatments are known to be associated with cardiovascular toxicity and increased risk in thromboembolic events, further trials are needed to define the benefits from treatment [102].

## 6. Endothelins and Nitric Oxide

After SAH, there is an imbalance between the vasodilator effects of nitric oxide (NO) and the vasoconstrictor effects mediated by endothelins, which also cause oxidative stress and inflammation. Endothelin-1 (END1) is a potent vasoconstrictor of cerebral vasculature and plays a critical role in vasospasm, also up-regulating VCAM-1 and ICAM-1 adhesion molecules’ expression [103,104]. END1 and its receptors—ENDRA and ENDRB—are involved in SAH complications, including vasospasm, DCI and functional outcome after bleeding. In the Cerebral Aneurysm Renin Angiotensin System (CARAS) study, common endothelin single-nucleotide polymorphisms (SNPs) had a strict relationship with the development of SAH. TG and TT alleles (genotype) of the END1 SNP (rs1800541) were tied to higher odds of developing SAH compared to controls. At the same time, a dominant effect emerged for the G allele, with CG and GG phenotypes at (rs5335) causing a four-fold increase in the risk of developing vasospasm [104]. Moreover, an interaction between cytokines and END1 expression and IL-6 also emerged, with the pharmaceutical blockage of IL-6 translating to lower END1 expression in staining tests [104]. As the role of END1 seems crucial to maintaining vasospasm, trials have been conducted to test the efficacy of clazosentan, an endothelin receptor antagonist, in vasospasm prevention after SAH. Despite the fact that the results were only marginally satisfactory, with clazosentan 5 mg/h found not to provide any benefit to patients on functional status, a 17% relative risk reduction was observed among those receiving clazosentan, suggesting that there might be room for personalized medicine [7]. Intercellular adhesion molecule (ICAM-1), bFGF, IL-7, IL-12p40 and MCP-4 variations over time have also been shown to significantly differ between SAH patients with good vs. bad clinical outcomes [66]. The increased concentration of ICAM-1 within 24 h was already known to be linked with the SAH severity and development of worse prognosis, and might also be a potential target for future interventions [38,66].

### 6.1. TLR4 and HMGB1 Association with Vasospasm and SAH

After SAH, damage-associated molecular patterns (DAMPs) are released in the subarachnoid space and bind to pattern recognition receptors (PRRs) expressed in central nervous system cells, including microglia, neurons, astrocytes, endothelial and smooth muscle cells. Toll-like receptors (TLRs) are one of the most well-studied PRRs, as they can sense various DAMPs, including cell lysates from red blood cells, high-mobility group box 1 (HMGB1) proteins, fibrinogen, heat shock proteins, matricellular protein tenascin C and other intracellular components of ruptured cells [105].

TLR4 has been found to play a key role in the inflammatory response after SAH and has been studied as a potential biomarker and therapeutic target [106]. The TLR4 pathway requires two extracellular binding partners, MD-2 and CD14, to activate the signal transduction events. The resulting cascade events are uniquely characterized by activations of two transcriptional nuclear factors (NF-κB and AP-1 mediated by MAPKs) through two distinct pathways: the MyD88-mediated “early phase”, which produces pro-inflammatory mediators, and the TRIF-dependent “late phase”, which induces the synthesis of the anti-inflammatory and anti-apoptotic interferon-β [106]. Thus, TLR4 in the late phase after its activation may be protective, and receptor antagonists in this phase may prevent recovery and regeneration.

TLR4 seems directly involved in EBI and vasospasm. In SAH mice models, tenascin-C knockout prevented neurological impairments, brain edema and BBB disruption through the inactivation of the TLR4/MAPKs pathway [107]. Moreover, the intra-cisternal injection of tenascin-C provoked neurological impairments and induced severe prolonged cerebral arterial constriction due to TLR4 up-regulation [108]. Microglial TLR4 seems to be essential for the development of vasospasm via TNF-α induction [60], a mechanism which may also directly roll out through MAPKs present in vascular smooth muscle cells [109]. What does TLR4 respond to after SAH?

HMGB1 is released in the extracellular space after SAH from necrotic cells or activated macrophages and acts like DAMP to ligate and activate TLR4. It is also released from vascular smooth muscle cells in the affected arterial walls after SAH and facilitates the further release of HMGB1 from neuronal nuclei, inducing a vicious cycle. Treatment with anti-HMGB1 antibody significantly suppressed the delayed vasospasms in a rat model of SAH [110] and reduced brain injury by preventing BBB disruption and inflammation [57].

In clinical studies, high levels of HMGB1 in the CSF and plasma of patients following SAH have been identified as possible biomarkers of neurological injury and predictors of vasospasm. In a longitudinal study including 303 SAH patients and 150 healthy subjects, high plasma levels of HMGB1 on admission were identified as independent predictors of in-hospital mortality, vasospasm and 1-year poor outcome [111] (Zhu et al., 2012). In a smaller sample study, including 30 people with SAH, higher levels of TLR4 within 24 h from SAH predicted DCI and 3-month poor neurological outcome [112]. Thus, TLR4 and its ligand HMGB1 may represent one of the most upstream components of innate immunity that is potentially “druggable” to prevent vasospasm and DCI.

### 6.2. Genetic Polymorphism and Risk of SAH Complications

As the occurrence of EBI, DCI and vasospasm is variable across patients with SAH, research has progressively investigated mutations and polymorphisms at sites other than END1, aiming to find a clue to the higher susceptibility to SAH complications [113,114]. An association between vasospasm and the SNPs of nitric oxide endothelial synthase (eNOS), haptoglobin and END1 receptor has been found in different studies [114,115].

A recent meta-analysis highlighted that the Haptoglobin 2-2 allele genotype was associated with an almost four-fold increase in the risk of vasospasm [114]. The C-allele of the eNOS SNP T786C (rs2070744) was found to be independently associated with a 3-fold higher risk of DCI [116]. eNOS intron VNTR a allele almost doubled the risk of clinical deterioration and DCI [114]. In addition to vasospasm, the eNOS T-786C SNP was also found to discriminate between small and large ruptured aneurysms, suggesting that this might be a relevant factor to consider in the long-term management of aneurysms [117].

As ryanodine receptors (RYRs) are deeply involved in the regulation of the luminal concentration of calcium in smooth muscle cells, with direct implications to vessel patency modulation, RYR1 variants were also investigated. In a single-center study, c.6178G > T, a genotype of RYR1, carried a 6-fold increase in risk for the development of symptomatic vasospasm [118].

Considering the other SNPs assessed in previous studies, those responsible for the asymmetric dimetilarginine and the high-mobility group box 1 (HMGB1) have been associated with DCI [119,120], whereas the SNPs of the angiotensin-converting enzyme seem to be related to DCI and poor neurological outcome [121].

## 7. Final Remarks

SAH carries high mortality and disability rates, which are substantially driven by complications. EBI and vasospasm are not rare entities after SAH, and despite being more frequent in more severe cases, they can also compromise the prognosis in less severe cases [1]. As immunological mechanisms have emerged as underlying these processes, treatments acting on the immune system are becoming more and more commonly tested for efficacy and safety in SAH patients.

Corticosteroids are drugs with broader actions on the immune system and have been investigated with the aim of a global immunomodulation in the acute phase after SAH. In a recent meta-analysis, a 50% reduction in rates of vasospasm emerged from clinical studies, with no significant heterogeneity across them. Such a result emerged from a small sample involved, suggesting that the effect size might be indeed reasonable to promote investigations [122]. Several antibodies are being tested in preclinical settings. An anti-HMGB1 monoclonal antibody has been shown to improve vasospasm in an animal model of SAH, with effects exerted in the very early phase [110]. Adalimumab, a TNF-α-targeted antibody, has been shown to exert a neuroprotective effect on SAH, consistently limiting vasospasm in animal models [123]. In clinical phase II studies, the subcutaneous administration of IL1RA controlled the rise in IL-6 and acute-phase proteins and conveyed a marginal increase in good prognosis rates, with no critical adverse events [71,124]. A phase III study on IL1RA is currently ongoing (NCT03249207), with results expected in late 2024 (Table 2).

The mounting evidence posits that immune inflammation, encompassing immune cells and biologically active substances, may take part in mechanisms behind various complications of SAH, including vasospasm. Inflammatory biomarkers exhibit a reasonable predictive ability for these events and furnish a critical window for prompt intervention. Although the efficacy and safety of immune-modulatory therapy necessitate further exploration, they represent a promising avenue to attenuate the alarming rates of mortality and disability associated with SAH, a stroke subtype that predominantly affects young adults and carries critical burdens for caregivers and society.

## Figures and Tables

**Table 1:** Immune cells involved in vasospasm after SAH.

Immune Cell	Course	Involvement in Vasospasm
CD45 lymphocytes	Increase in peripheral blood and cerebral spinal fluid	Activate and produce cytokines that promote inflammation and may contribute to vasospasm. CD45 lymphocytes may also interact with endothelial cells, promoting the formation of microthrombi and contributing to vasospasm.
Macrophages	Gradual increase in CSF after SAH	Critical for hemosiderin product scavenging, which may reduce oxidative stress and inflammation associated with vasospasm. Macrophages may also secrete pro-inflammatory cytokines, promoting inflammation and contributing to vasospasm.
Microglia	Local activation; release pro-inflammatory cytokines and chemokines	May contribute to inflammation and tissue damage and also phagocytose debris and promote tissue repair. Microglia may also interact with neurons and astrocytes, promoting inflammation and contributing to vasospasm.
Neutrophils	Early infiltration after SAH is associated with increased risk of vasospasm	Release of toxic enzymes and reactive oxygen species may contribute to vascular injury and inflammation, increasing the risk of developing vasospasm. Neutrophils may also interact with platelets, contributing to the formation of microthrombi and vasospasm.
NK cells	Increase both early and after initial vasospasm stage	May mediate direct toxicity to neurons and contribute to tissue damage. NK cells may also release cytokines that promote inflammation and contribute to vasospasm.
T cells in aneurysmal wall	Transition into CD4+ IL-17-producing cells	Increases production of IL-17, which promotes inflammation and may contribute to vasospasm. T cells may also secrete interferon-gamma, promoting inflammation and contributing to vasospasm.
T regulatory cells	Decrease in number and function after SAH	Reduced immunosuppressive function may contribute to inflammation and vasospasm.

**Table 2:** Promising drugs targeting immune system for vasospasm prevention after SAH.

Drug	Action	Stage and Results
Anakinra	Interleukin-1 receptor antagonist	Phase II trial reported a substantial limitation in IL6 levels with treatment; Phase III trial (NCT03249207)
Bevacizumab	Anti-VEGF antibody	Attenuated cerebral vasospasm after intraperitoneal injection in animal models
Clazosentan	Endothelin receptor antagonist	Reduces the risk of DCI and vasospasm in adults with SAH (results from observational and trial data available)
Dexamethasone	Synthetic adrenal corticosteroid that shows potent anti-inflammatory activity	Phase III trial (NCT05132920)
Nafamostat	Serin protease inhibitor with endothelial protective effects	Improved neurological outcome and reduced MMP-9 levels after SAH in animal models
Satralizumab	Monoclonal antibody that blocks the interleukin-6 (IL-6) receptor	Phase I trial (NCT05727657)
Adalimumab	Monoclonal antibody against TNF-α	Attenuated basilar vasospasm and limited arterial wall thickness in basilar artery vasospasm in animal models
Satralizumab	Monoclonal antibody that blocks the interleukin-6 (IL-6) receptor	Phase I trial (NCT05727657)

## Data Availability

Data can be shared upon reasonable request to the corresponding author.
